# Periostin is critical for improving the therapeutic properties of adipocyte-derived stem cells

**DOI:** 10.1186/s13287-015-0215-x

**Published:** 2015-11-04

**Authors:** Theresa Chow, Ian M. Rogers

**Affiliations:** Women’s and Infants’ Health, Lunenfeld Tanenbaum Research Institute, Mount Sinai Hospital and University of Toronto, 60 Murray Street, Box 40, Toronto, M5T 3L9 ON Canada; Department of Physiology, Medical Sciences Building, University of Toronto, 3rd Floor, 1 King’s College Circle, Toronto, M5S 1A8 ON Canada; Department of Obstetrics and Gynecology, Faculty of Medicine, University of Toronto, 123 Edward Street, Suite 1200, Toronto, M5G 1E2 ON Canada

## Abstract

Periostin is a matricellular protein that is reactivated during tissue damage and repair and has been shown to be a critical regulator of multiple biological pathways involved in the repair of tissue after myocardial infarction, peripheral vascular disease, and skin wounds. The tissue repair properties attributed to periostin make it an ideal candidate to enhance the therapeutic properties of donor cells such as mesenchymal stem cells from adipocyte tissue. In a recent article in *Stem Cell Research & Therapy*, Qin et al. demonstrated enhanced therapeutic properties of adipocyte-derived stem cells by genetically engineering them to express periostin.

Mesenchymal stromal or stem cells (MSCs) from different tissue sources have a proven track record of enhancing tissue repair through the direct contribution to new tissue by engrafting and differentiating into muscle, endothelial cells, adipocytes, and bone. MSCs also have a powerful paracrine signaling system resulting in the enhancement of endogenous repair mechanisms. Despite the advantages of cell-based therapy, one of the common problems facing the use of cells for tissue repair is the low survival rate of the donor cells. In a recent article in *Stem Cell Research & Therapy*, Qin et al. modified adipose-derived stem cells (ADSCs) to improve their survival [1]. Normally, donor cells are subjected to host inflammation, hypoxia, and mechanical stress, all of which lead to apoptosis and cell survival rates of less than 1 % [2]. There have been attempts to overcome these adverse conditions through the addition of genes, mRNA, or proteins to modify the donor cells or by modifying the host environment with the goal of enhancing donor cell survival. Alternatively, cell carriers, in the form of hydrogels or the addition of extracellular matrix (ECM) and matricellular proteins that offer both mechanical and biological support, have demonstrated enhanced donor cell survival and increased tissue repair [3–5], but more improvements are still required to meet the needs of patients.

Qin et al. modified ADSCs to express the periostin gene by using lentivirus vectors and determined that the modified ADSCs had superior tissue repair properties compared with unmodified control ADSCs by using a model of peripheral vascular disease (PVD) [1]. Periostin is a matricellular protein that has multiple effects because of its ability to activate several biological pathways. Periostin is expressed embryonically but is re-expressed in adults after tissue damage and during tissue repair. Because periostin is secreted and is a component of the ECM, it has a role in inducing cell survival, proliferation, migration, and adhesion to the ECM. In the context of tissue repair, Qin et al. showed that periostin-secreting ADSCs had a greater contribution to tissue repair compared with unmodified cells because they survived better in the hypoxic environment generated by damaged tissue and were able to engraft and differentiate into endothelial and muscle cells. Furthermore, the periostin-producing ADSCs demonstrated an enhancement of their paracrine signaling-mediated tissue repair mechanism [1].

Tissue repair, whether in the context of PVD [6], skin wounds [7], spinal cord injury [8], or cardiac ischemia [9], follows a similar healing process. The initiating event is followed by inflammation that includes the migration of macrophages to remove cell debris and the infiltration of neutrophils and fibroblasts. The fibroblasts lay down new ECM that permits the further migration of cells into the wound area. Fibrocytes and myofibrocytes remodel the new ECM, resulting in an organized protein network that provides a foundation for tissue repair, including engraftment of MSCs, which can differentiate and contribute to new tissue or induce repair through the induction of endogenous cells through a paracrine signaling mechanism [10]. The eventual replacement of fibroblasts with tissue-appropriate cells results in proper tissue architecture instead of scar tissue formation. For most tissue repair situations, the production of scar tissue is the end result, and tissue remodeling to produce normal tissue takes years. In older patients or patients with severe tissue damage, the scar tissue may be permanent. Treatment with exogenous MSCs does result in reduced scarring because of the enhancement of the tissue repair process.

Adipose tissue, bone marrow, and umbilical cord tissues are all commonly used sources of MSCs to treat tissue damage. Qin et al. used ADSCs instead of bone marrow-derived MSCs, cells which share similar properties, because adipose tissue collection is minimally invasive, making them more clinically applicable. Qin et al. also chose lentivirus to introduce the periostin gene into the cells. Although the use of lentivirus faces more stringent safety measures compared with using other non-permanent methods to modify cells, lentivirus vectors offer advantages such as stable transfection of the cells, resulting in long-term periostin production at the site of cell transplantation. Furthermore, lentiviruses do not induce an immune reaction in the host, and lentivirus-modified cells are currently being used in multiple clinical trials (www.ClinicalTrials.gov). Previous studies have used non-integrating plasmids to deliver genes [11], mRNA transfection [12], or the direct injection of protein into the damaged tissue, thus bypassing the use of cells completely [4]. However, both plasmid and mRNA transfections are inefficient and have a short half-life, and the direct injection of protein results in the diffusion and degradation of the protein.

Periostin has a central role in tissue repair, acting at the nexus of multiple biological pathways. Inflammation induces transforming growth factor beta 1 (TGFβ1) and tumor necrosis factor alpha (TNFα), which in turn regulate critical ECM protein and matricellular proteins, including periostin, heat shock protein (hsp), and fibronectin [13]. Periostin then activates the phosphatidylinositol-3-OH kinase (PI3K) pathway and the downstream kinase Akt. PI3K/Akt signaling regulates several functions, including cell survival, growth, cycling, and migration [3]. Studies that engineered MSCs to express AKT [14], PI3K [3], or Hsp [15] confirm that all of these proteins have a role in wound healing. By genetically engineering ADSCs to express periostin, a central regulator of multiple biological pathways involved in tissue repair, Qin et al. make an important advancement in cell therapy that is applicable to multiple disease systems besides PVD, such as myocardial infarction and diabetic skin ulcers [1].

## Abbreviations

ADSC: Adipose-derived stem cell
ECM: Extracellular matrix
hsp: Heat shock protein
MSC: Mesenchymal stromal or stem cell
PI3K: Phosphatidylinositol-3-OH kinase
PVD: Peripheral vascular disease
